# α-Synuclein and Polyunsaturated Fatty Acids: Molecular Basis of the Interaction and Implication in Neurodegeneration

**DOI:** 10.3390/molecules23071531

**Published:** 2018-06-25

**Authors:** Chiara Fecchio, Luana Palazzi, Patrizia Polverino de Laureto

**Affiliations:** 1 Department of Biomedical Sciences, University of Padova, 35131 Padova, Italy; chiara.fecchio@bio.unipd.it; 2 Department of Pharmaceutical and Pharmacological Sciences, CRIBI, University of Padova, 35131 Padova, Italy; luana.palazzi@unipd.it

**Keywords:** alpha-synuclein, Parkinson’s disease, polyunsaturated fatty acids, neurodegeneration

## Abstract

α-Synuclein (α-syn) is a 140-amino acid protein, the physiological function of which has yet to be clarified. It is involved in several neurodegenerative disorders, and the interaction of the protein with brain lipids plays an important role in the pathogenesis of Parkinson’s disease (PD). Polyunsaturated fatty acids (PUFA) are highly abundant in the brain where they play critical roles in neuronal membrane fluidity and permeability, serve as energy reserves and function as second messengers in cell signaling. PUFA concentration and composition in the brain are altered with age when also an increase of lipid peroxidation is observed. Considering that PD is clearly correlated with oxidative stress, PUFA abundance and composition became of great interest in neurodegeneration studies because of PUFA’s high propensity to oxidize. The high levels of the PUFA docosahexaenoic acid (DHA) in brain areas containing α-syn inclusions in patients with PD further support the hypothesis of possible interactions between α-syn and DHA. Additionally, a possible functional role of α-syn in sequestering the early peroxidation products of fatty acids was recently proposed. Here, we provide an overview of the current knowledge regarding the molecular interactions between α-syn and fatty acids and the effect exerted by the protein on their oxidative state. We highlight recent findings supporting a neuroprotective role of the protein, linking α-syn, altered lipid composition in neurodegenerative disorders and PD development.

## 1. Introduction

Parkinson’s disease (PD) is a progressive, neurodegenerative disorder, characterized by severe motor symptoms, such as tremor, difficulty in movements and rigidity. PD has a multifactorial etiology and constitutes the second most common neurodegenerative disease, after Alzheimer’s disease (AD). Reactive oxygen species (ROS) level imbalance, mitochondrial dysfunction and loss of dopamine-producer neurons in the *substantia nigra* are some of the most common disease hallmarks. The pathological distinctive elements of PD are represented by the Lewy bodies (LB) and Lewy neuritis (LN), ubiquinated protein inclusions mainly composed of the presynaptic protein α-synuclein (α-syn) [1]. Mutations in α-syn were identified in families with hereditary PD [2,3,4,5,6,7]. Both in familial and in sporadic PD, the protein is present as an insoluble filamentous aggregate, known as a fibril, with specific morphology and structure, and α-syn dysfunction appears a critical determinant for the development of the disease [8].

Despite the intense investigations on the structure and putative function of α-syn, the detailed relationship between the protein and the onset of PD is not clarified yet [9]. The ability of α-syn to interact with membranes and more in general with lipids has made this topic the target of numerous studies. The key event in neurons that leads to PD and neurodegeneration is not defined, and several pathways that underlie both genetic and sporadic forms of the disorder are possible. Fatty acids, especially brain polyunsaturated fatty acids (PUFAs) and their relationship with α-syn, are stimulating considerable attention. PUFAs serve as an energy reservoir, contribute to signaling pathways and regulate many processes in the brain such as neurotransmission, cell survival, mood and cognition. However, in conditions of oxidative stress, as well as in patients affected by PD, the neuronal levels of PUFAs in a non-esterified form were found higher than normal [10] and, anyway, altered in various neurological disorder [11]. In particular, the levels of those PUFA-derived substances, representing the markers of oxidized lipids, were found to be ten-fold higher than normal in the *substantia nigra* of patients affected by PD [12].

In this review, the molecular features of the interaction between α-syn and brain PUFAs, especially arachidonic acid (AA) and docosahexaenoic acid (DHA), are discussed in the context of PD. We and other hypothesize that the protein/PUFA molecular ratio in neurons is a discriminant factor, and increases in the levels of either certain PUFAs or α-syn monomers in the cytoplasm can produce different effects in physiological and pathological conditions [13,14,15]. Under physiological conditions, α-syn can actively participate in the control of oxidative homeostasis of the intracellular environment, protecting the free fatty acids from oxidation and maintaining their correct level. Environmental factors can affect this equilibrium, by either increasing oxidative stress or changing DHA concentration. Since protein oligomerization has been observed upon exposure to PUFAs and because oligomers might represent the most toxic species, the increased DHA concentration in the cell could exacerbate toxicity rather than having a protective role [13,16,17].

Studies of drugs targeting PUFAs are underway as a novel approach for the exaltation of their beneficial effects on neurodegeneration, but probably further investigations are required to clarify the implication of brain PUFAs in health and disease and the link with α-syn.

## 2. Brain PUFAs: Structure, Metabolism and Function

### 2.1. Lipid Composition and Metabolism in the Brain

The brain contains a high concentration and variety of lipids, especially fatty acids (FAs), second only to adipose tissue. The composition of FAs in the brain is very complex, and AA and DHA represent the most abundant polyunsaturated FAs (PUFAs) [18,19]. In particular, DHA reaches 10% of total FAs in the brain [20]. Generally, phospholipids are present in the membrane as glycerophospholipids composed of mixed saturated and unsaturated FAs, with one and up to six double bonds. PUFAs are classified as omega-3 (n-3) and omega-6 (n-6) on the basis of the location of the last double bond relative to the terminal methyl end of the molecule (Table 1).

Very long FAs are synthesized in liver and reach brain through circulation [16]. Different mechanisms have been reported to describe FAs crossing of the brain blood barrier, including FA/albumin complexes, circulating lipoproteins or diffusion without dedicated transporters [16,21]. FAs can then pass the plasma membrane by a flip-flop mechanism, whereas their diffusion outside the cells is prevented by acyl-CoA derivatization. Acyl-CoA synthetases are the first enzymes, among several, which are implicated in FAs metabolism and regulation [16]. Long-chain-fatty-acyl-CoA synthases (ACSLs) specifically activate DHA and AA, of which the majority will then be esterified to phospholipids immediately upon lipids entering the cell [22]. PUFAs, such as AA and DHA, usually occupy the *sn*-2 position of the glycerol backbone, while saturated FAs normally are in *sn*-1 [23]. This specificity is conserved also in the dedicated enzymes for their de-esterification, since phospholipase A-1 (PLA-1) acts on fatty acids in *sn*-1 and phospholipase A-2 (PLA-2) is specific for *sn*-2 lipids, such as DHA and AA [24]. Moreover, it has been demonstrated that DHA turnover can be modulated without affecting AA metabolism, indicating a selective regulation among these two FAs. In particular, calcium-independent PLA-2 is selective for DHA release [25]. Upon PLA-2-induced release, around 90% of PUFAs are esterified back to phospholipids, while the remaining portion may be metabolized to active derivatives through the action of different enzymes such as cyclooxygenases (COX), lipoxygenases (LOX) and cytochrome P450 [26].

### 2.2. The Functions of PUFAs in the Brain

**Figure 1:**
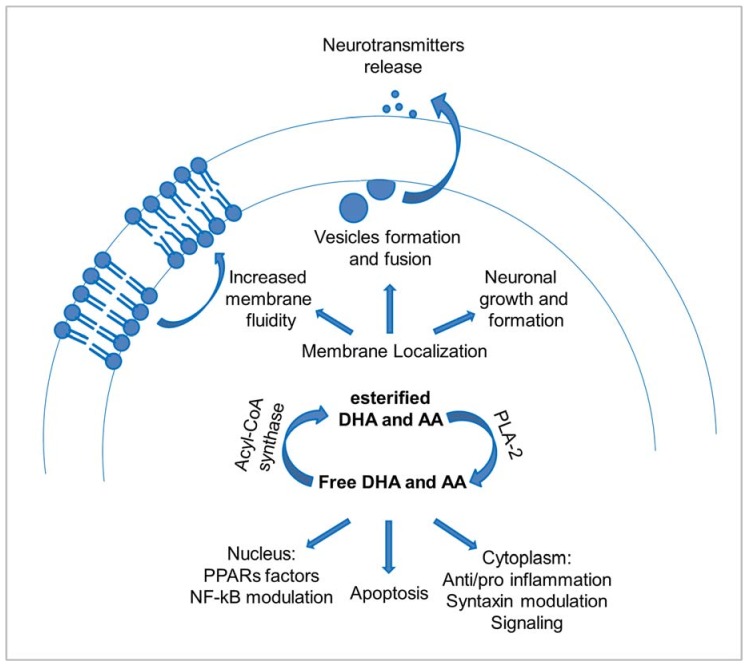
DHA and AA functions in neurons. Schematic representation of the different essential roles of DHA and AA and their metabolites, according to their esterified or free state.

Among the different FAs present in the brain, omega-6 (n-6) and omega-3 (n-3) are essential; therefore, they must be obtained from food [28]. These PUFAs are abundant in neuronal membranes and exert several important effects (Figure 1). Notably, DHA and AA deficiencies lead to severe problems in the brains, since n-3 such as DHA is required for brain development and function, while n-6 such as AA yields essentials metabolic products. Increasing evidence suggests that a balanced dietary intake of these PUFAs has beneficial effects in maintaining the health state of the brain [16]. In particular, dietary intake of DHA strongly influences DHA levels in different tissues, in a faster way than it happens for AA [29,30].

The presence of PUFAs guarantees membrane fluidity and plasticity, providing a more dynamic environment due to the high number of double bonds, even if in a different extent between n-3 and n-6 fatty acids [10]. As a consequence, not only the presence of PUFAs, but also the balance between n-3 and n-6 PUFAs influences different processes on membranes, such as trafficking and vesicle budding and fusion [28].

PUFAs act also on vesicles formation and fusion in an active manner, contributing to an efficient release of neurotransmitters and hormones or neurite outgrowth [23,31]. Indeed, PUFAs activate with high specificity a protein localized on the plasma membrane, syntaxin, pushing it to enter the SNARE complex [32], which is essential for vesicle trafficking [31,33,34]. When phospholipases induce the release of PUFAs, typically occupying the *sn*-2 position in phospholipids, they become available to directly contribute to many processes or to be converted into eicosanoids [35]. PUFAs can activate syntaxin, and PUFA metabolites can either activate or inhibit vesicle fusion processes [32].

Among other important functions, PUFAs act also on the apoptosis pathway [36]. Apoptosis is a tightly-controlled process bringing cells to death without causing an inflammatory response. A recognized signal triggering of this process is the accumulation of phospholipase-released free PUFAs [37]. On the other side, also cell proliferation is influenced by lipids [38].

Finally, a major role in brain health involves the pro- or anti-inflammatory effect of molecules deriving from the catabolism of n-6 or n-3 PUFAs, respectively [28]. Several actions derived from PUFA metabolites have opposite effects depending on their n-3 or n-6 FA derivation [28]. DHA and AA, de-esterified by PLA-2 enzymes, can be metabolized through different pathways, but generally, metabolites of n-3, such as DHA or eicosapentaenoic acid (EPA), show anti-inflammatory properties, while those deriving from AA show opposite effects [39,40]. Notably, COX2, which facilitates the formation of prostaglandin E2 from AA, is expressed at a higher level in neurons than in any other tissue. AA may also be converted to other metabolites, usually conducible to pro-inflammatory activity [41]. On the contrary, DHA metabolites are less clearly identified, but the main known products include 17S-hydroxy-DHA, 14-hydroxy-DHA and LOX-induced resolvin D5, maresin 1 and neuroprotectin D1 [42]. These molecules, at variance with AA products, are reported to act as “pro-resolving” mediators [26], since they are involved in an active mechanism of inflammation resolution. The discovery of mediators with anti-inflammatory or “pro-resolving” effects derived from n-3 PUFAs highlighted the presence of active pathways for inflammation resolution other than a “dilution effect”, able to actively compete with AA metabolites [26], underlining the importance of such protective mechanisms in the brain.

To exert their functions, n-3 metabolites have been reported to inhibit *NF-κB* (nuclear factor kappa-light-chain-enhancer of activated B cells), thus controlling several cytokines [43], and to bind the peroxisome proliferator-activated receptor (PPAR) family of transcription factors more efficiently than n-6-derived metabolites [44]. Given their different and sometimes opposite effects, the importance of a strict controlled ratio between n-6 and n-3 accumulation and metabolism in the brain becomes clear.

### 2.3. PUFAs in Aging and Disease

The correlation of PUFA composition with brain health is suggested by the fact that it is altered in an age-dependent manner [45]: specifically, a decrease in n-6 PUFAs has been reported, while n-3 PUFAs tend to increase [46]. During aging, intensified lipid clearance and catabolism are required, due to neuron death and decrease in brain volume [47]. Above all, the main factor with aging resides in the increase of lipid peroxidation, promoting neurodegenerative diseases such as AD or PD [48]. The brain is the main oxygen consumer organ in the body, accounting for around 23% of the total in adulthood [49]. Moreover, the brain has a high-energy demand to ensure neuronal transmission, transport and protein synthesis, and it mainly relies on glycolysis [50]. These facts make the brain vulnerable to oxidative damage, considering the elevated presence of easily-oxidizable PUFAs and strong need for glucose metabolism. Notably, all PUFAs are subject to attack by oxygen because of the presence of double bonds, but DHA is especially at risk due to its five bis-allylic sites that make it highly prone to oxidation [51]. Due to the high concentration of DHA in the brain, high metabolic rates and high levels of reactive oxygen species, efficient antioxidant mechanisms are mandatory, and indeed a considerable amount of energy within the brain is devoted to this goal [18], with different systems developed to protect DHA from oxidation and to scavenge and replace damaged DHA [51]. Recycling of FAs within membrane phospholipids is probably the most efficient mechanism to remove damaged lipids, with PLA-2 and ACSL enzymes able, in rodents, to recycle each day up to 100% of membranes. This great effort requires a highly-dedicated energy expenditure, which has been calculated to be around 5% of ATP consumption in the brain [52]. This precarious equilibrium has been reported to be altered in aging [45], when the normal protective systems may fail to counteract the oxidative damage, as well as in the development of neurodegenerative diseases, where it may represent a major triggering factor [41]. In a recent work, we suggested that also α-syn may have a role in protecting neurons from oxidized DHA products, as will be discussed below [15].

## 3. α-Synuclein: Structure and Physiological Role

### 3.1. The Structure and Function of α-Synuclein

**Figure 2:**
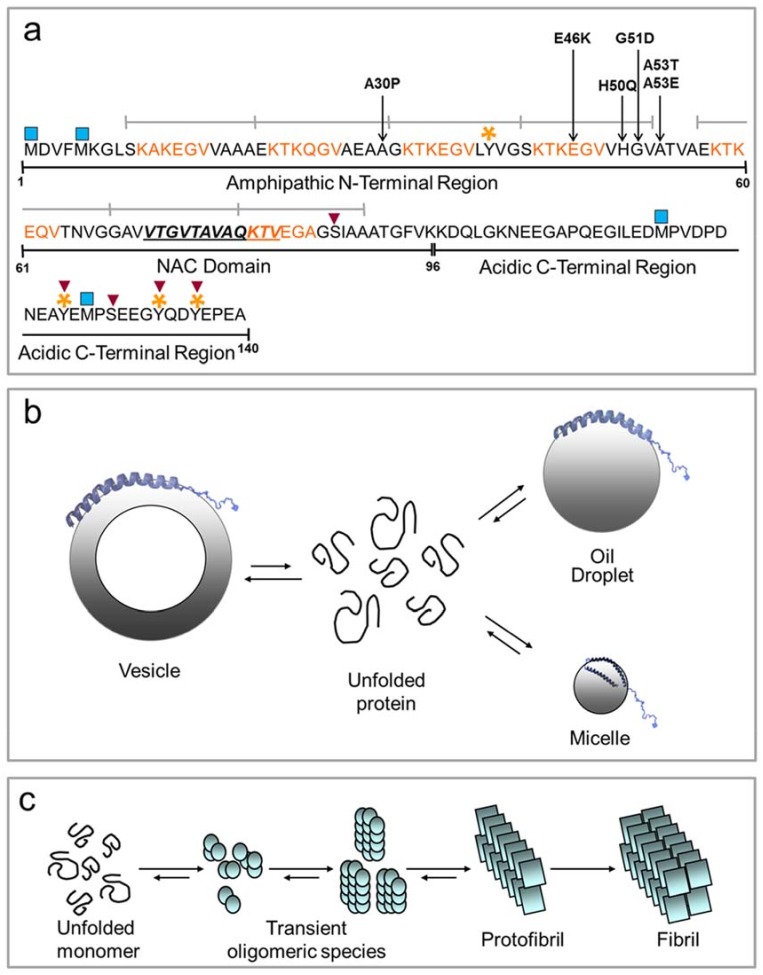
Scheme of the structure of α-syn. (**a**) The three major domains of the protein are indicated: the N-terminal region involved in lipid and membrane binding (1–60), the NAC domain (61–95) responsible for aggregation properties of the protein and the acidic C-terminal region, able to transiently interact with the N-terminal and the NAC domains, modulating the aggregation propensity of the protein (96–140). The 11-amino acid imperfect repeats are indicated by grey lines. The highly conserved amino acid motifs are colored in orange. The 12 residues (71–82) essential for amyloid aggregation are also highlighted. The main pathological missense mutations are located in the N-terminal region. The post-translational modifications are indicated by symbols: blue square, oxidation; red inverse triangle, phosphorylation; orange stars, nitration; (**b**) In the presence of vesicles, micelles and oil droplets, the N-terminal domain of α-syn acquires an α-helical secondary structure. The unbound protein is unfolded (curved lines); (**c**) Schematic mechanism of amyloid aggregation of α-syn. The process is presumably a step-wise formation of larger multimeric protein species, starting from the monomeric unfolded protein.

α-Syn is a 140-residue protein, highly expressed in the central nervous system, particularly at the presynaptic nerve terminals [53,54]. In addition, α-syn was found to be ubiquitously expressed throughout the body [55,56,57]. It is the major component of LB, the cytoplasmic proteinaceous aggregates pathognomonic for PD [8]. Its sequence can be divided into three parts (Figure 2a), the amino-terminal (1–60), the central region (61–95) and the carboxy-terminal (96–140). The amphipathic lysine-rich amino terminus, containing four of the seven 11-amino acid imperfect sequence repeats, with a highly conserved hexameric motif (KTKEGV), has a crucial role in modulating the interaction with membranes and lipids and resembles the amphipathic helices present in apolipoproteins [58,59]. The central region (61–95), characterized by hydrophobic residues responsible for α-syn aggregation, is called NAC (non-amyloid-β component), and it is present in the amyloid plaques [60]. In β-synuclein, another member of the synuclein family that shares 78% of homology with α-syn, the NAC region is partially deleted, and this protein acts as an inhibitor of aggregation [61,62]. Moreover, the NAC region contains the other three 11-amino acid imperfect repeats. The carboxyl-terminal region (residues 96–140) is enriched in acidic and proline residues, and it is suggested to play an important role in modulating the aggregation properties of the protein, its localization and interaction with metals, small molecules and proteins [63,64,65].

All pathological mutations of α-syn, responsible for the familial form of PD, are localized in the N-terminus region. These lead to early-onset (A30P, E46K, A53T, G51D) or late-onset (H50Q) forms of the disease [2,3,4,5,6,7]. The missense mutations G51D, A53E and A30P are responsible for defective interaction of α-syn with membranes and lipids [66,67,68]. More recently, H50Q was reported to be implicated in PD by increasing the aggregation rate of α-syn [5,6,7]. Interestingly, the point mutants tend to form stable β-sheets, thus exacerbating the formation of toxic oligomers, protofibrils and fibrils [69]. Therefore, it is believed that the missense α-syn mutations cause PD through a toxic gain of function [69], and LB may represent an attempt to purge the cell of toxic damaged α-syn [70].

Beyond the genetic mutations, α-syn is subjected to posttranslational modifications (PTMs) that are mainly implicated in prompting or decreasing the rate of fibril formation and in modulating membrane interaction [71]. Some of these modifications are represented by phosphorylation, occurring mostly at serine 87 and 129 and tyrosine 125, 133 and 135 residues [72,73], acetylation at the N-terminal [74], oxidation of the methionine (Met) residues and nitration at the tyrosine, especially the 39 and 125 residues [75,76]. SUMOylation, glycation, glycosylation and proteolysis were also reported for α-syn, even if the position or the biological and physiological implications are not well clarified yet [77,78,79,80]. Of interest, Met oxidations (especially Met1 and Met5) seem to play an important role in modulating α-syn membrane binding [81]. Moreover, in vivo, these Met can be reduced by methionine sulfoxide reductase. Exploiting this physiological reversibility, α-syn can contribute to the protection of membranes from oxidative damage [81]. Further, α-syn containing oxidized Met forms stable oligomers and inhibits α-syn fibrillation [82]. By using a combined approach of proteolysis and mass spectrometry, we showed that in the presence of PUFAs (AA and DHA), α-syn can be chemically modified. In particular, the early radical products of DHA and AA autoxidation can react with α-syn, producing a covalent modification on the protein. The histidine at position 50 seems to be the major target of this reaction. These data suggested a possible physiological role for the protein, that is a sequestering ability to capture free radicals. On this basis, we proposed that the protein may play a neuroprotective role in neurons in response to oxidative stress, eventually preventing oxidation of PUFAs [15]. Consistently, Zhu et al. [83] have previously found that α-syn can prevent lipid oxidation also when bound to membranes.

The association of α-syn to membrane and lipid has been widely studied, since several pieces of evidence correlate the protein’s physiological function, still poorly understood, with lipids. Indeed, α-syn seems to be implicated in processes correlated with synaptic vesicle homeostasis, contributing to synaptic vesicles formation, fusion, regulation, plasticity and recycling [71,82,84,85,86,87]. Other evidence links α-syn to the modulation of dopamine biosynthesis and dopamine release from pre-synaptic vesicles [88,89]. A role for α-syn was proposed in the assembly and disassembly of the SNARE complex, reporting that α-syn seems to act as a chaperone, necessary for vesicle exocytosis and neurotransmission [90]. Furthermore, as specified above, the interaction between α-syn and cell membrane phospholipids and its sequence similarity with apolipoprotein have led to the hypothesis of a role for the protein as a lipid transporter. In fact, α-syn does not simply interact with membranes, but is able to remodel them [91]. Further, it was shown that α-syn and PUFAs’ interaction affects endocytosis and vesicle recycling in both neuronal and non-neuronal cells and specifically activates synaptic vesicle recycling after neuronal stimulation by enhancing clathrin-mediated endocytosis [92]. A role for α-syn in brain lipid metabolism has also been suggested, since fatty acid uptake and metabolism appear affected in the absence of the protein [93,94,95]. In particular, these authors have shown that the deletion of the SNCA gene induces the opposite effects on DHA and AA brain metabolism and suggested that the increase in incorporation and turnover of DHA in specific brain phospholipids is the result of metabolic compensation for the reduced incorporation and turnover of AA [95].

### 3.2. The Physiological and Pathological Folding of α-Synuclein

The topic about the α-syn secondary structure in vivo is still debated. α-Syn, expressed in *Escherichia coli*, was found to be monomeric and natively unfolded [96], but it can acquire an α-helical structure upon interaction with membranes and lipids [58,63], or β-sheet conformation, when it is embedded in amyloid fibrils [97]. Under physiological conditions, α-syn was reported to be in equilibrium between membrane-bound and unbound states (Figure 2b). The binding of α-syn to membranes is related to their chemical properties and preferentially occurs with membranes rich in acidic lipids [98], with highly curved and with small unilamellar vesicles (SUVs) [58]. Moreover, α-syn binds membranes mainly composed of long PUFA tails and inositol as the head group [99,100]. Upon lipid binding, α-syn can adopt different types of α-helical structure, as seen by using different biophysical techniques. Ulmer et al. [101] found that, as a result of SDS-micelle binding, α-syn can form two anti-parallel curved α-helices (Val3-Val37 and Lys45-Thr92) connected by a well-ordered linker, whereas the ending C-terminal tail remains highly mobile. By using a site-directed spin labeling and electronic paramagnetic resonance EPR-based approach, it was demonstrated that in the presence of lipid bilayers, the first 90 amino acids of α-syn adopt an extended curved α-helix, with a periodicity of 11/3, parallel to the membrane, where lysine and glutamic residues interact with the zwitterionic headgroups, while the uncharged residues interact with the hydrophobic region [102]. Other studies report the formation of a unique helical structure upon α-syn binding to SUVs, rod-like SDS micelles and lipid bicelles [103]. Ferreon et al. [104] showed a model of interaction of α-syn with SUVs and SDS micelles in which an interplay exists between a broken and an extended α-helix. However, the structure of α-syn unbound states is still not clarified. Recently, endogenous α-syn was isolated from neuronal and non-neuronal cell lines by non-denaturing techniques, and it was shown that the protein was physiologically present in a tetrameric form rich in α-helical structure [105]. This was previously observed also for α-syn expressed in *E. coli* [106]. This tetramer was shown to have low propensity to aggregate into amyloid fibrils. In contrast, other studies confirm the natively unfolded nature of α-syn [107,108]. Nonetheless, an ensemble of conformers of α-syn has been shown to exist in vivo [109,110].

In vitro and in vivo, α-syn can undergo amyloid fibril formation, with a typical cross-β sheet structure by a nucleation-dependent mechanism [97,111]. The nucleation process is the rate-limiting step in fibril formation that gives rise to the production of soluble oligomers, proto-fibrils and finally mature amyloid fibrils (Figure 2c). Recently, several lines of evidence indicate that the soluble oligomers, rather than the mature fibrils, may be the toxic species associated with PD [112], and LB/LN are a protective and inert forms in which fibrils converge [113].

## 4. Molecular Interaction between α-Synuclein and PUFAs

### 4.1. Fatty Acids Induce Changes in α-Synuclein Secondary Structure

**Figure 3:**
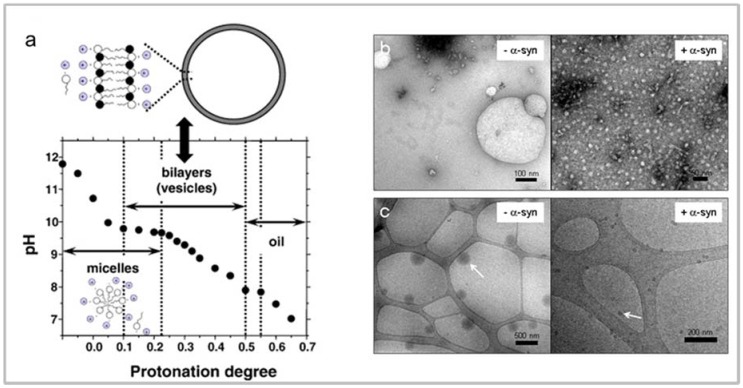
Biophysical properties of PUFAs. Titration curve for oleic acid/sodium oleate. (**a**) The plot indicates the regions for the formation of micelles, vesicles and oil droplets as a function of pH; Negative staining (**b**) and cryo (**c**) electron microscopy images of samples of DHA in the absence (− α-syn) and in the presence (+α-syn) of the protein. The arrows indicate the oil droplets. Taken, with permission, from [113,120].

Upon exposure to PUFAs (DHA and AA), α-syn acquires an α-helical secondary structure with a rapid equilibrium between its free and the lipid-bound form [114,115,116,117]. This conformational transition is a function of PUFA concentration and follows a two-state model [114]. The study of this interaction is very challenging, since the protein and FAs mutually and dynamically affect their physical properties. It has been shown that for this interaction, a prerequisite is the presence of at least one double bond in the fatty acid. Palmitic and arachidic acids, for example, do not induce the formation of α-helical structure in α-syn, while oleic acid (OA), a MUFA, does [114,115,116,117]. Two factors modulate the protein/PUFA interaction in vitro: (i) the self-assembly/aggregation state of the FA, regulated in turn by its critical aggregative concentration (CAC) in solution; and (ii) the molecular ratio between the FA and the protein. PUFAs exhibit a high tendency to self-assemble, in vitro, being composed of amphiphilic chains. This process strongly depends on PUFAs’ concentration, as well as on the media ionic strength, temperature and pH [116,118,119,120]. More specifically, the PUFAs are present as a mixture of a neutral form and a negatively-charged form as a function of pH [121]. In diluted solution and above a critical concentration, PUFAs can dynamically form micelles, vesicles and oil droplets as a function of the ratio between the ionized and non-ionized species (Figure 3a). Micelles are the dominant aggregation species at pH > 9; oil droplets form above pH 8; whereas PUFAs preferentially form vesicles in the pH range of 8–9 [119]. These lipid aggregates show dynamic nature, wide size variability and different surface characteristics, as seen by transmission electron (TEM) and cryo-transmission electronic microscopy (cryo-TEM) (Figure 3b), strongly affecting the modality of interaction with proteins [114,116,117,122,123].

**Figure 4:**
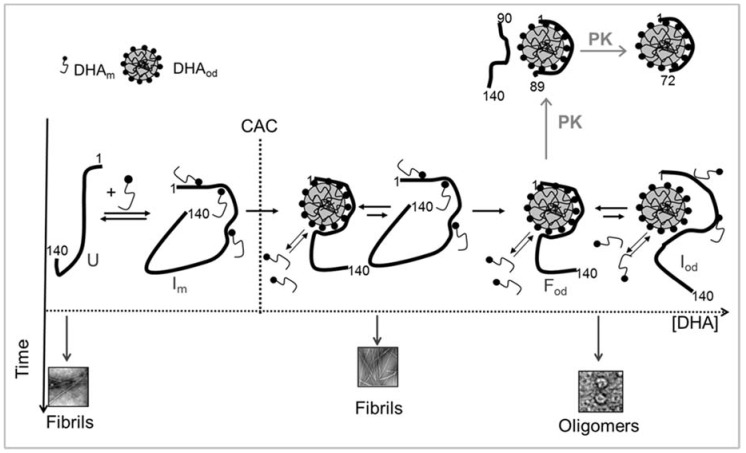
Conformational transition and aggregation of α-syn in the presence of DHA. The DHA self-assembly process as a function of the critical aggregative concentration (CAC) in the presence of the protein is shown. DHA_m_ and DHA_od_ represent the fatty acid monomer and the oil droplet containing DHA, respectively; U, the unfolded free protein; I_m_, the protein in the presence of non-saturating concentration of DHA; F_od_, the protein in the presence of oil droplets; I_od_, the species in equilibrium with the lipid-bound protein. The early and subsequent proteolytic events by proteinase K (PK) are shown. In the absence of DHA, α-syn, unfolded in solution (U), undergoes structural transition and forms amyloid-like fibrils. In the presence of DHA (P/DHA ≥ 10), free α-syn molecules are in equilibrium with the bound ones. Under this condition, transient intermediates form (I_m_), and these species evolve in amyloid-like fibrils, as free α-syn molecules are available. The aggregation occurs in a shorter time than in the absence of the fatty acid. In the presence of DHA (P/DHA ≥ 50), all α-syn molecules are bound to the fatty acid (F_od_), acquiring an α-helical conformation, and the formation of stable oligomers occurs.

The molecular ratio between α-syn and PUFA (P/PUFA) seems to play a key role in their dynamic interplay, affecting the equilibrium between the bound and free forms of the protein (Figure 4). Employing a non-saturating DHA concentration (i.e., P/DHA molar ratio of 1:10), about 65% of the free protein molecules are in equilibrium with the bound fraction. Circular dichroism studies show that, under these conditions, the protein adopts a partly-folded state, substantially α-helical. At a P/DHA molar ratio of 1:50, almost all protein molecules are bound to DHA; the fraction of free molecules is negligible; and the protein conformation is completely converted into an α-helical structure [114]. Accordingly, by using SUVs, Galvagnion et al. [124] have shown that a high lipid: α-syn ratio implies that essentially all the protein molecules are bound to the vesicle surface and are in helical conformation. The presence of free protein molecules in equilibrium with the aggregated FA can have variable consequences on protein aggregation and oligomerization, as discussed below.

Interestingly, the association of α-syn with MUFAs and PUFAs in the form of vesicles, micelles or oil droplets affects their stability and integrity, leading to a destabilization of these self-aggregation products (Figure 3b,c). Generally, a variation in both PUFA aggregates’ size and surface properties has been observed, generating species with a smaller diameter and lowering the FA concentration necessary to form aggregates [114,116,117]. This phenomenon is correlated with the availability of the protein since, when all the protein molecules are bound to the FA, the free FA molecules are not prevented from forming aggregates of various sizes, corroborating the opinion that the protein/PUFAs’ interaction is a mutual and dynamic process. This ability to disperse PUFA aggregates leads one to speculate a putative protective function of α-syn in the cellular environment [13].

A debated question is which type of interaction occurs between α-syn and FAs at the molecular level. The presence of the imperfect repeats at the α-syn N-terminus and some similarity with the amino acid sequences of fatty acid binding proteins (FABPs) have previously suggested that α-syn might be a member of the intracellular FABP family or might work as an FA transporter [58,115,125]. Although α-syn is able to bind FA in a manner reminiscent of FABPs, by using a combined approach of NMR and TEM, Lücke et al. [125] concluded that the protein does not likely act as an intracellular fatty acid carrier, and the ability to bind to negatively-charged membranes is one of its intrinsic, possibly physiological, properties [116]. Limited proteolysis experiments were shown to be a useful tool to map the α-syn/DHA complex at the molecular level, demonstrating that the N-terminal region is engaged in the presence of DHA [114]. At a DHA/protein molar ratio of 50, in which all α-syn molecules are bound to the FA, α-syn region 89–102 resulted in being flexible and proteases sensitive. The produced N-terminal 1–89 and then 1–72 fragments, resistant to further proteolysis, accumulated in the reaction mixture and retained α-helical structure, suggesting that these species derive from the digestion of the protein bound to DHA and are very important for the interaction (Figure 4). We also analyzed the propensity of α-syn isolated fragments to interact with DHA, confirming that the interaction with the fatty acid is mediated by the N-terminal portion of α-syn [126]. It has been also demonstrated that the α-syn N-terminal region from residues 2–60 is responsible and essential for the binding to PUFAs and for the FA-induced oligomerization of the protein [127]. We showed that the peptide corresponding to the sequence 1–52 is able to trigger the binding of α-syn to DHA oil droplets and to exhibit the same ability of the full length protein to change the fatty acid aggregative properties [126]. To reinforce these considerations, it is important to underline the general role of the α-syn N-terminus for its membrane-assisted binding and folding [109,128]. It has been suggested that the short sequence comprising residues 1–25 is responsible for the nucleation of the cooperative coil/helix transition of the adjacent large protein domains in the presence of lipid [109].

In conclusion, the molecular interaction between α-syn and PUFA seems to be mediated by two biophysical criteria, of electrostatic and hydrophobic nature. The positively-charged N-terminal domain of α-syn, which is essential for the cooperative formation of helical domains in the protein, is able to recognize the net negative lipid surface charge of PUFA [109]. The hydrophobic, fluid lipid core of polyunsaturated acyl chains favors the immersion of the protein into the PUFA layer [116].

### 4.2. Fatty Acid-Induced α-Synuclein Oligomerization

The exposure of α-syn to PUFAs (or vesicles containing PUFAs) induces its fast oligomerization and in some cases also fibrillation both in vivo and in vitro [17,100,122,123,129]. Perrin et al. [100] have shown that also the PD-associated variants A30P and A53T of α-syn oligomerize in vitro upon exposure to vesicles containing PUFA at physiological concentrations of protein and phospholipids. The protein aggregation is a subsequent event, but strictly correlated with PUFA binding. A mutant deleted of the N-terminus, essential for binding, is not able to oligomerize, when transfected in HEK293 cells [127]. Oligomers induced by PUFAs exhibit different structural and biological properties compared to other type of α-syn oligomers found in brain or generated in vitro [130]. The oligomeric species of α-syn grown in the presence of DHA in vitro resulted in being toxic in cultured dopaminergic cells [122]. These oligomers, containing a partial α-helical structure, in cellular membrane-mimetic and cell model systems, seem to exert their cytotoxic effect through transient alteration of membrane permeability [131].

The effects of PUFAs on α-syn aggregation in vivo are quite complex, but in the majority of studies, a strong correlation between α-syn oligomers’ appearance and increase in PUFA levels in the brain was found. In particular, PUFA levels have been found elevated in PD and LB disease (DLB) brain-soluble fractions and in neuronal cells overexpressing α-syn or its pathological mutants. An increased PUFA content induces an increase in membrane fluidity in neurons overexpressing α-syn, while in α-syn, genetically deleted mice, both PUFA content and membrane fluidity seem to be decreased. On the basis of these observations, Sharon et al. [13] concluded that α-syn and PUFAs’ interaction could physiologically contribute to regulating the neuronal PUFA levels. Moreover, exposure of neuronal cell lines to PUFAs increases the levels of α-syn oligomers, suggesting that PUFAs in vivo could promote the formation of α-syn-soluble oligomers as precursors of the insoluble aggregates associated with neurodegeneration [17]. Further, the same group in another work provided evidence that PUFA-induced soluble oligomers exhibit cytotoxicity in the neuronal cell line, whereas PUFA-induced inclusions, successively formed, may be protective [132]. In oligodendroglia (OLN) cell lines, overexpressing the α-syn A53T mutant, the formation of α-syn aggregates resembling the inclusion observed in neurodegeneration upon exposure to DHA was observed, which was incorporated into cell membranes, as well as oxidative stress [133].

Recently, Iljina et al. [129] have proposed that AA-induced α-syn oligomers could potentially form in vivo, and this could represent a species in which the protein has acquired an α-helical structure, in equilibrium with the unfolded monomer protein, prone to aggregation. The potential function of this AA-protein complex could be protective, leading to the stabilization of the protein in a structured form resistant to further aggregation. In the context of PD, this may contribute to the reduction in cytotoxicity associated with PUFAs [134]. In conclusion, the accumulation of α-syn, including as soluble oligomers, is associated with alterations in brain PUFA composition. Specifically, such protein accumulation occurs especially in those fractions of brains with PD and DLB, where also certain long-chain PUFAs (especially DHA) are found at more than normal levels. This raises the possibility that brain PUFA content could be therapeutically downregulated, reducing the natural tendency of oligomerization of α-syn, losing its deleterious effects [13,17].

In vitro studies have shown to be very useful to rationalize the effects of PUFA on α-syn oligomerization. Both the structure of fatty acids and the PUFA/protein ratio affect the kinetics and the type of aggregation, as well as the morphology of the grown aggregates. Indeed, PUFAs, but not saturated FA, can induce the oligomerization and aggregation of α-syn [17,132], suggesting that this phenomenon is correlated with the specific property of PUFA to form micelles, vesicles or other aggregated species as a function of their concentration. Moreover, it was shown that in the presence of an excess of lipid in which all protein molecules are bound to the fatty acid, the protein undergoes oligomerization [117,122,124]. At low PUFA/protein ratios, when free monomer is present in solution, the bound ones serve as starting nuclei for further aggregation, leading to fibril formation [117,122]. The rate of the nucleation reaction is at least three orders of magnitude greater than that occurring in bulk solution, due to the high local concentration of protein molecules at the surface of the vesicle/micelle/droplet and to their likely ability to explore conformations that may favor primary nucleation [124].

Around 5–10% of PD cases are linked to genetic mutations [135], among which at least four known missense mutations are on the SNCA gene coding for α-syn [136]. The most common variants are A30P, E46K, H50Q and A53T [2,3,4,5,6,7], and interestingly, the mutations fall in the region, which plays a role in the interaction with lipids [66,67,68]. α-Syn seems to interact with phospholipids through two different binding mode, called SL1 and SL2 [128]. These states differ for the length of the interacting sequence, involving residues 3–25 for SL1 and up to residue 100 for SL2 state. All the studied variants populate the SL1 state, which anchors the protein to the lipid while causing the hydrophobic NAC region to remain unfolded and thus more prone to aggregation. These differences may be linked to specific variations of the amino acid properties, as for example the presence of a proline for A30P that may disrupt the secondary structure. From a pathological point of view, the loss of α-syn-lipid interaction impairs α-syn scavenger function, while on the other side, it favors the protein oligomerization [128]. Data about how these variants interact with PUFAs are lacking. We observed a similar aggregation propensity of variants A30P, E46K and A53T upon interaction with DHA, acquiring an α-helix in a lipid dose-dependent manner and forming stable oligomers as those observed for the wild-type protein with a protein-lipid ratio of 1:50 [15,137]. However, considering the demonstrated protein-PUFAs interplay and the suggested scavenger role of α-syn, it would be interesting to deepen these aspects in the presence of natural gene mutations, since structure-determined differences similar to those observed for phospholipids are likely to emerge.

## 5. Implication on Neurodegeneration

Despite the progresses made in the knowledge of the structure and function of α-syn, its role in the onset of PD and more in general in neurodegeneration is far from being understood. The interplay between α-syn and PUFAs could provide a key link, since the ability of monomeric α-syn to interact with free and aggregated PUFAs has different consequences both in physiological and pathological conditions.

Our data suggested that the physiological and pathological effects of α-syn are determined by distinct regions of the primary structure. For its role as a scavenger of oxygen radicals, lysine, methionine and histidine residues are involved, as well as the structural interaction with PUFAs is an essential requisite [15]. In fact, we have interpreted the covalent modifications occurring on α-syn after DHA exposure as a protective response to PUFAs’ peroxidation [15], according to the recent hypothesis that α-syn may act as scavenger of ROS in healthy cells [138]. The presence of α-syn in synaptic terminals and its ability to bind to plasma membranes, lipid rafts, inner nuclear membrane and mitochondrial membrane are correlated with the physiological function of the protein [71]. In a simplified scheme, α-syn might interact with free PUFAs, released from neuronal membranes, before their turn-over processes would be activated. Indeed, in human brain, DHA and AA, as repeatedly underlined, are the main fatty acids in membrane phospholipids [139], which are essential for the maintenance of the fluidity and permeability of neuronal membranes. PUFAs and their mediators regulate several processes within the brain, such as neurotransmission, cell survival and neuroinflammation, and thereby mood and cognition [11]. They are released from membrane phospholipids in the cytosol by PLA-2. Then, PUFAs follow their natural destiny of recycling or degradation [10]. Changes in the activity of PLA-2 enzymes, altered PUFA metabolism and/or associated phenomena could induce an increase of the DHA level, with a series of consequences, which ultimately lead to neuronal damage [10]. However, it seems that DHA levels depend on the age, diet, gender and genotype of genes involved in the endogenous synthesis of DHA and are in relation to the initiation and progression of neurodegenerative disorders [140,141]. It was also observed that levels of DHA in the non-esterified form are elevated in patients affected by PD or LB dementia [13]. However, the question is still debated, since it has also been reported that deficits in DHA are associated with cognitive decline during aging and in neurodegenerative disease [139].

In this picture, we considered the protein/DHA ratio as a discriminant factor between the biological function and the pathological consequences. As seen so far, on the one hand, α-syn might have a protective role due to its scavenging activity on lipid oxidation [15,83,138] and to its ability to modulate and maintain the PUFAs’ correct levels in the brain [13]. This occurs in physiological conditions, in which the normal amount of oxidized DHA can be scavenged by α-syn, together with other concurring protective cellular mechanisms. An increase in the PUFA/α-syn ratio could lead to an increase in α-syn aggregation as a result of enhanced α-syn binding to PUFA oil droplets, but increased binding could also interfere with the formation of β-sheet-rich oligomers of α-syn by favoring α-helix formation and by diluting α-syn molecules on the oil droplet surface.

On the other hand, it could be possible that under certain pathogenic conditions mediated by either increasing oxidative stress (aging, PD) and changing DHA/AA concentration, α-syn/PUFA interaction may have negative prerogatives, and the same protein-lipid interaction causes α-syn to form toxic oligomers upon incubation. The formation of stable toxic oligomers is observed when α-syn is incubated in the presence of a high concentration of DHA and AA [17,122,131,132], but also by oxidation of α-syn 4 Met residues, that in turn could be promoted under conditions of oxidative stress [142]. It could be possible that lipid oxidative species increase to an extent that does not allow the normal biological turnover of α-syn, leading to the accumulation of modified protein and increasing the propensity of its oligomerization. Since oligomers seem to be the toxic species rather than fibrils, we believe that the increasing DHA concentration in the cell could worsen toxicity rather than having a protective role.

## Figures and Tables

**Table 1:** Main fatty acids in human pre-frontal cortex. The most common brain fatty acids are grouped according to their unsaturation degree, reporting common names and chemical classification.

Classification ^a^ C:D n-X	Common Name ^b^	Pre-Frontal Cortex FAs (%) ^c^	Structure ^d^
**SFA**			
12:0	Lauric acid (LA)	36	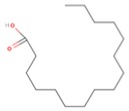
14:0	Myristic acid (MA)		
16:0	Palmitic acid (PA)		
18:0	Stearic acid (SA)		
24:0	Lignoceric acid (LCA)		
**MUFA**			
18:1 n-9	Oleic acid (OA)	28	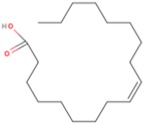
24:1 n-9	Nervonic acid (NA)		
16:1 n-7	Palmitoleic acid (POA)		
17:1 n-7	Heptadecenoic acid (HA)		
18:1 n-7	Vaccenic acid (VA)		
**PUFA**			
22:6 n-3	Docosahexaenoic acid (DHA)	15–18 DHA ~ 13.5	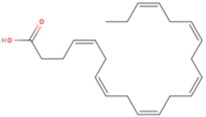
18:3 n-3	α-Linolenic acid (ALA)		
22:5 n-3	Docosapentaenoic acid (n-3DPA)		
20:5 n-3	Eicosapentaenoic acid (EPA)		
18:2 n-6	Linoleic acid (LNA)	17 AA ~ 9	
20:4 n-6	Arachidonic acid (AA)		
22:5 n-6	Docosapentaenoic acid (n-6DPA)		

^a^ Classification according to carbon chain length (C) and number of double bonds (D), as well as starting position of double bonds from the last methyl group (n-X). ^b^ SFA, saturated fatty acid, MUFA, monounsaturated fatty acid, PUFA, polyunsaturated fatty acid. ^c^ From [27]. ^d^ Structures of one fatty acid representative of each class: palmitic acid for SFAs, oleic acid for MUFAs and docosahexaenoic acid for PUFAs.
